# In this issue of Current Oncology

**Published:** 2006-08

**Authors:** M. McLean

Many of the newer clinical studies proposed for the management of high-risk prostate cancer include the use of chemotherapy in addition to androgen deprivation. This natural development follows on from publications endorsing the value of chemotherapy in metastatic prostate cancer. In 1994, a Canadian group was the first to show that, by using mitoxantrone combined with steroids, a significant number of patients could improve their quality of life; and in 2004, the results from clinical trials of docetaxel were published showing for the first time that the lives of men affected by hormone-refractory prostate cancer could be prolonged. One of these published reports came from a study carried out at the Princess Margaret Hospital in Toronto, where the original research using mitoxantrone and steroids had been carried out. The introduction of docetaxel into prostate cancer treatment is a milestone, and it is natural that this agent be explored in the adjuvant setting. Given these advances in care for men with prostate cancer, we are pleased to enclose with this issue of *Current Oncology* a new patient guide titled *Living with Prostate Cancer.* The guide discusses the newer treatment strategies in hormone refractory disease, including the use of docetaxel.

The World Health Organization continues to report new cases of human infection with avian influenza in the Far East, and although the next influenza pandemic may not involve the H5N1 strain, a pandemic is nonetheless predicted. Various national, regional, and institutional plans have been and continue to be developed to mitigate the consequences of such an event. In this issue of the journal, the unique needs of patients with cancer during a pandemic are discussed.

The recent increased waiting times for adjuvant radiation in the province of Ontario led to breast cancer patients from one Canadian cancer treatment centre being referred to two centres in the United States. That situation provided a unique opportunity to compare radiation practices between the two jurisdictions. Significant and quite substantial variations in prescribing patterns were seen, likely to impact on patient convenience and resource utilization.

Another article in this issue is concerned with identifying strategies that might promote a healthy diet. Despite an extensive search effort, few beneficial dissemination strategies were identified. Much of the evidence turned out to be descriptive rather than evaluative, suggesting that controlled studies are needed to evaluate dissemination strategies and to compare dissemination and diffusion strategies with varying messages and varying target audiences.

Finally, a new section, Updates and Developments in Oncology, which benefits from the editorial guidance of Richard Ablin, appears in this issue. The authors of the inaugural review in this series—“Targeting transforming growth factor β to enhance cancer immunotherapy”—explain that, to develop effective cancer therapy modalities, an understanding of the biologic effects that tumour-derived factors have on immune cells is essential.

